# Comments to the Editor: opinions on “Causal effects of primary biliary cholangitis on irritable bowel syndrome: a Mendelian randomization study”

**DOI:** 10.1097/JS9.0000000000002759

**Published:** 2025-06-27

**Authors:** Ping Zhao, Qiuwan Han, Xu Qiu

**Affiliations:** a Department of Anesthesiology, Hangzhou Xiaoshan District Orthopedics Hospital of Traditional Chinese Medicine, Hangzhou, Zhejiang, China; b The Fourth Clinical School of Medicine, Zhejiang Chinese Medical University, Hangzhou, China

*Dear Editor*,

The use of artificial intelligence (AI) in research and literature is increasing, but we still need to exercise moderation in its application^[1]^. Recently, I read a study, this study employs two-sample Mendelian randomization (MR) techniques for the first time to explore the potential causal relationship between primary biliary cholangitis (PBC) and irritable bowel syndrome (IBS), a topic of significant clinical relevance. The research design strictly adheres to the three core assumptions of MR and includes comprehensive sensitivity analyses, such as MR-Egger intercept tests, Cochran’s Q tests, and leave-one-out analyses, which are notable strengths of this article. The findings suggest that PBC may increase the risk of IBS (IVW OR = 1.010887, P = 0.04136), opening new avenues for investigating the “gut-liver axis” mechanism. However, several points could be further refined to enhance the robustness of these conclusions:

## Consideration of confounding factors

The selected single nucleotide polymorphisms (SNPs) may violate the exclusion restriction assumption due to insufficient consideration of other factors that could lead to IBS, such as anxiety/depression and childhood trauma. Selection Criteria for Instrumental Variables: In this study, only SNP-exposure association strength (p<5 × 10^–8^) and linkage disequilibrium were considered when selecting instrumental variables (IVs). However, SNPs with direct genetic associations with IBS were not excluded from analysis. For example, some SNPs linked to PBC might directly affect IBS risk through non-PBC pathways like immune regulation or bile acid metabolism, introducing confounding bias. It is recommended that phenotype-wide association studies (PheWAS) be conducted to identify SNPs without direct associations with IBS as IVs. As emphasized by Burgess et al., violations of exclusion restrictions are common sources of bias in MR studies; thus verifying SNP specificity through cross-phenotype databases like GWAS Catalog is essential^[2]^. Reliability Concerns Regarding IVW Method Results: The reliability of results from IVW methods is questionable, as it only showed weak positive significance (P = 0.04136), while four other methods, including MR-Egger and weighted median, did not reach significance (P>0.05). This inconsistency indicates that IVW results may be affected by weak instrument bias or residual pleiotropy effects.

## More importantly, no correction for multiple testing was performed in this study

We applied FDR correction on five reported MR analysis outcomes in this paper—IVW, MR-Egger, weighted median, simple mode, and weighted mode—and found that after FDR adjustment the minimum p-value obtained via IVW was Pₐd_j_ = 0.2068 (>0.05), indicating that exposure-outcome associations are no longer significant. Bowden et al. (2016) noted that when conclusions across various MR methods are inconsistent; caution should be exercised in interpreting significance from IVW results while correcting for multiple testing is crucial for controlling false-positive rates^[3]^.

## Exploration of heterogeneity sources and subgroup effects

While acknowledging limitations regarding GWAS data being confined primarily within European populations, the discussion fails addressing potential impacts arising from genetic backgrounds or environmental factors(such as gender/age).Subgroup analyses or inclusion considering gene-environment interactions would provide valuable insights into these aspects. For instance Kurilshikov et al.’s (2021) multivariable-MR approach corrected confounders revealing population heterogeneity concerning gut microbiome-disease correlations^[4]^. Moreover existing literature indicates an association between sex differences influencing incidence rates among individuals diagnosed with IBD where females exhibit markedly higher prevalence than males; unfortunately, this aspect wasn’t separately discussed which represents an oversight.

## Deepening clinical significance

Referencing Mars et al.’s (2020) proposed “gut-liver-brain axis” theory can facilitate discussions surrounding how hepatic injury resulting from PBC influences occurrences linked towards development mechanisms underlying IBS cases providing directionality towards future mechanistic investigations^[5]^. Optimization Of Data Utilization: Integrating multi-source GWAS datasets (e.g.FinnGen) to validate findings will enhance statistical power overall.

In summary, this research provides preliminary evidence for causal links between PBC and IBS. However, limitations related to selection strategies for instruments used, the absence of corrections during multiple testing, and inconsistencies in methodologies necessitate strengthening the robustness of the conclusions. By incorporating additional verification measures focused on specific SNPs and conducting reverse-MR assessments while addressing multiplicity issues, we can significantly enhance the credibility of this inquiry. This will help clarify the roles played by gut-liver axes in both disease entities and yield precise targets for clinical comorbidity management moving forward.

## Data Availability

Not applicable.
